# A Systematic Review on the Risk Modulators of Myocardial Infarction in the “Young”—Implications of Lipoprotein (a)

**DOI:** 10.3390/ijms24065927

**Published:** 2023-03-21

**Authors:** Cristian Stătescu, Larisa Anghel, Laura-Cătălina Benchea, Bogdan-Sorin Tudurachi, Andreea Leonte, Alexandra Zăvoi, Ioana Mădălina Zota, Cristina Prisacariu, Rodica Radu, Ionela-Lăcrămioara Șerban, Radu Andy Sascău

**Affiliations:** 1 Internal Medicine Department, ”Grigore T. Popa” University of Medicine and Pharmacy, 700503 Iași, Romania; cristian.statescu@umfiasi.ro (C.S.); benchea.laura-catalina@d.umfiasi.ro (L.-C.B.); bogdan-sorin.tudurachi@d.umfiasi.ro (B.-S.T.); ioana-madalina.chiorescu@umfiasi.ro (I.M.Z.); cristina.prisacariu@umfiasi.ro (C.P.); rodica.radu@umfiasi.ro (R.R.); radu.sascau@umfiasi.ro (R.A.S.); 2 Cardiology Department, Cardiovascular Diseases Institute “Prof. Dr. George I. M. Georgescu”, 700503 Iași, Romania; leonteandreea32@gmail.com (A.L.); alexandra.zavoi@gmail.com (A.Z.); 3 Physiology Department, “Grigore T. Popa” University of Medicine and Pharmacy, 700503 Iași, Romania; ionela.serban@umfiasi.ro

**Keywords:** lipoprotein (a), low-density lipoprotein, acute myocardial infarction, “young” patients, risk factors

## Abstract

The presence of a myocardial infarction at a younger age is of special interest, considering the psychological and socioeconomic impact, as well as long-term morbidity and mortality. However, this group has a unique risk profile, with less traditional cardiovascular risk factors that are not well studied. This systematic review aims to evaluate traditional risk factors of myocardial infarction in the “young”, highlighting the clinical implications of lipoprotein (a). We performed a comprehensive search using Preferred Reporting Items for Systematic Reviews and Meta-analyses (PRISMA) standards; we systematically searched the PubMed, EMBASE, and Science Direct Scopus databases, using the terms: “myocardial infarction”, “young”, “lipoprotein (a)”, “low-density lipoprotein”, “risk factors”. The search identified 334 articles which were screened, and, at the end, 9 original research articles regarding the implications of lipoprotein (a) in myocardial infarction in the “young” were included in the qualitative synthesis. Elevated lipoprotein (a) levels were independently associated with an increased risk of coronary artery disease, especially in young patients, where this risk increased by threefold. Thus, it is recommended to measure the lipoprotein (a) levels in individuals with suspected familial hypercholesterolaemia or with premature atherosclerotic cardiovascular disease and no other identifiable risk factors, in order to identify patients who might benefit from a more intensive therapeutic approach and follow-up.

## 1. Introduction

Cardiovascular diseases (CVD), and, in particular, coronary artery disease (CAD) continue to represent one of the most important causes of morbidity and mortality all around the world. Globally, it is estimated that deaths caused by cardiovascular diseases will significantly increase in the coming years, from approximately 17.3 million deaths per year today, to more than 23 million by 2030 [1,2,3]. Thus, coronary heart disease continues to be a major health problem, and while there are a lot of studies available for adults, there is a scarcity of data on myocardial infarction in the “young”. This may cause an underestimation of the importance of CAD in young patients, in whom both the short-and long-term consequences can be devastating. Thus, an acute coronary syndrome in young patients may have an important impact on the patient’s psychology, socioeconomic and professional burden, family, as well as long-term morbidity and mortality. The incidence of CAD tends to increase from the fourth decade of life; therefore, most studies refer to persons younger than 40–45 years [4]. To date, there is no universally accepted age cut-off when defining “young”, regarding the acute myocardial infarction (AMI). The age varies from ≤40 [5,6] to ≤55 years [6], and most of the studies suggest 45 years old as a cut-off [7,8]. Considering that there is no universally accepted cut-off age to define the young patients, in this article, we will use the one applied by each author of the cited articles. The epidemiology of coronary artery disease also shows geographic variability. South Asian patients appear to be more prone to CAD and AMI at an early age. A study of 877 patients in India with CAD revealed that one third were <45 years old at the time of initial diagnosis [9]. Regarding the trends in acute myocardial infarction by race and ethnicity, in a recent study, Chi and co-workers observed a decline in acute myocardial infarction hospitalization incidence rates across all race/ethnic groups during a 15-year period (2000 to 2014). The declines were similar for most race/ethnic groups; however, Black individuals experienced an important decline in acute myocardial infarction rates during the period 2000 to 2009, and this slowed during the period 2010 to 2014. Although acute myocardial incidence rates narrowed between Black and White groups, and between Asian or Pacific Islander and Hispanic groups, differences persist [10]. Unfortunately, nowadays, there are only few data regarding the risk factors of myocardial infarction in young patients. Traditional cardiovascular risk factors have an important role, but there are only few data regarding the importance of other newer risk factors in these young patients, such as lipoprotein (a) [Lp(a)] or those who activate inflammatory pathways. One of the inflammatory conditions associated with a high risk of atherosclerotic disease is the periodontitis, an inflammation of the tissues surrounding the tooth, which are infiltrated by neutrophils, macrophages, and activated lymphocytes. The most important two hypotheses regarding the mechanisms by which the periodontitis affects the blood vessel wall are the direct impact of the bacteria and their toxins on the vessel wall, and the cytokines and inflammatory mediators being released during the periodontal inflammation [11]. SARS-CoV2 infection induces an inflammatory state associated with a higher immune response and an instability of the atherosclerotic plaques, which can cause acute coronary syndromes. Thus, the inflammation inside the atherosclerotic plaques can increase the production of collagenase by macrophages, which reduces the thickness of the fibrous cap and increases the risk of plaque rupture and acute coronary events. In addition, SARS-CoV2 infection is associated with a high risk of coagulation abnormalities and coronary thrombosis [12]. However, the implications of periodontitis and SARS-CoV2 infection on the initiation and progression of atherosclerosis are not clear yet, and need to be further investigated. In the last years, knowledge about the implications of Lp(a) in atherosclerotic cardiovascular disease have increased substantially. Lp(a) has some pleiotropic effects on atherosclerotic cardiovascular disease; it promotes atherosclerosis due to its low-density lipoprotein cholesterol (LDLc) moiety, promotes thrombosis due to its similarity with plasminogen, and promotes arterial wall inflammation due to the oxidized phospholipids [1,10]. Thus, targeting Lp(a) seems to be at least as important as LDLc reduction, even in patients with LDLc values on target. Therefore, in August 2022, the European Atherosclerosis Society published the second consensus statement highlighting the causal association between Lp(a) concentration and cardiovascular outcomes, and provided clinical guidance for testing and treating elevated lipoprotein (a) levels [1]. In the first part of this paper, we reviewed the traditional risk factors of myocardial infarction in the “young”, while, in the second part, we highlighted the clinical implications of lipoprotein (a).

## 2. Methods

This review was carried out according to the Preferred Reporting Items for Systematic Reviews and Meta-analyses (PRISMA) standards. The studies regarding the implications of lipoprotein (a) in young patients were selected by performing a systematic search of PubMed, EMBASE, and Science Direct Scopus from database inception until January 2023. The search terms were “myocardial infarction”, “young”, “lipoprotein (a)”, “low-density lipoprotein”, “risk factors”, and these terms were searched in the title, abstract, and keywords. The bibliographies of all located papers were examined in order to identify relevant literature. According to the PRISMA standards, a methodological appraisal of each study was conducted, to further avoid possible biases. The following inclusion criteria were used: (1) original research articles, (2) studies coordinated by independent research groups, (3) implications of lipoprotein (a) in myocardial infarction in the “young”, and (4) only papers written in English. Non-English papers, animal-based studies, abstracts, editorials, case reports, and reviews were excluded. This study did not involve human subjects and was exempt from institutional review board approval.

**Figure 1:**
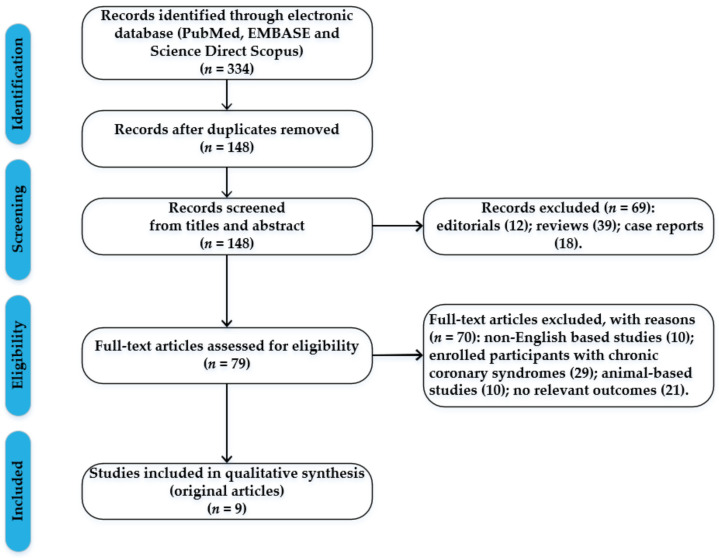
Flow chart with the process of article selection.

The search identified 334 articles, which were screened to exclude duplicates. The resulting 148 articles were then screened based on their abstract, which left 77 articles for further evaluation. These articles were then carefully evaluated, considering the main aims of the review. At the end, this evaluation left 9 original research articles regarding the implications of lipoprotein (a) in myocardial infarction in the “young” that were included in qualitative synthesis. Figure 1 illustrates our search strategy.

## 3. Results and Discussion

### 3.1. Myocardial Infarction Related to Traditional Cardiovascular Risk Factors in Young Patients

#### 3.1.1. Arterial Hypertension

Certain processes connect atherosclerotic disease with elevated blood pressure. The endothelial dysfunction, arterial wall remodelling, and the formation of atherosclerotic lesions are caused by a complicated interaction of a genetic predisposition, aberrant vasoreactivity, and vessel wall shear stress, in combination with neurohormonal activation [13]. Younger individuals have a distinct clinical presentation of CAD than older patients. Thus, young patients have a lower incidence of several established risk factors, shorter ischaemic time, and reduced mortality. Thomas et al. [14] showed that younger participants had a reduced prevalence of hypertension (14.2% vs. 28.3%; *p* < 0.001) in comparison to the older ones, in a cohort ST-segment elevation myocardial infarction (STEMI) research study on 2420 patients (591 of whom were under 45 years old). In addition, Yagel and co-workers observed that the prevalence of hypertension in the initial ACS incident was low, only in 20% of males below 40 and females below 50 years old, but the most important risk factor for recurrent cardiovascular events was hypertension (*p* = 0.0048) [15]. In addition, a retrospective study revealed that the prevalence of untreated hypertension was higher in patients <55 years (40.4 percent) than in those between 55 and <70 years (27.2%) (*p* = 0.007) [16]. Hypertension was found to be an independent risk factor for multivessel disease in young ACS patients (male and female ≤ 45 years) (OR 3.63, 95% CI 1.88–7.01, *p* < 0.001). Multivessel disease was diagnosed in about 40.1% of young patients with STEMI and was associated with a poor outcome compared to single-vessel disease (38% vs. 25,1%, *p* < 0.01) [17].

#### 3.1.2. Smoking

Smoking is still one of the greatest and most frequent risk factors among young MI patients [18]. According to Singh et al., in STEMI patients, smoking (37.6%) was shown to be the most prevalent risk factor for young STEMI subjects (patients under 45 years), followed by diabetes mellitus (16.8%), and hypertension (16%) [19]. In addition, smoking seems to be a particularly strong predictor of future MI in the young (all *p* < 0.05) [20]. Similarly, additional studies showed that young AMI patients (18 to 44 years of age) had higher rates of smoking (both cigarettes and water pipe smoking), along with hypertension and dyslipidaemia, with more than 70% of them having at least one risk factor [21,22,23].

#### 3.1.3. Diabetes Mellitus

Diabetes mellitus (DM) is a significant cardiovascular risk factor that causes an accelerated and severe atherosclerotic process [24]. Winter et al. [25] discovered that type 2 DM was one of the most common risk factors, along with smoking and hypertension, after studying 102 AMI survivors (aged ≤ 40 years). DM was also the greatest predictor of outcomes among traditional risk variables with a crude hazard ratio 2.36 ratios (HR) (95% CI, 1.07–5.28, *p* = 0.036). The recent retrospective research conducted on patients under the age of 50 who had their first AMI discovered that 20% of young MI patients had diabetes at the time of their first hospitalization. In addition, diabetes was linked to increased long-term all cause (HR 1.65, *p* = 0.008) and cardiovascular mortality (HR 2.10, *p* = 0.004) rates in these patients [26]. Early onset type 1 diabetes is associated with an overall hazard ratio for AMI of 30.95 (17.59–54.45) for those diagnosed in the age range 0–10 years, with the risk levels being 90 times higher for women [27]. Supplementary, according to the research by Bęćkowski et al. [28], diabetes was the strongest predictor of ACS in women ≤45 years of age, with a sixfold increase in risk (OR 6.66, 95% CI 3.47–12.74) along with hypertension (OR 4.30, 95% CI 3.42–5.38), hypercholesterolaemia (OR 3.45; 95% CI 2.60–4.29), and smoking (OR 1.63, 95% CI 1.34–1.98).

#### 3.1.4. Obesity

The incidence of obesity among young people has increased. Yandrapalli et al., discovered that obesity was the risk factor with the biggest increase in frequency among young people, indicating a 98% increase from 2005 to 2015 [21]. In a study involving 2739 men aged 18–44 years hospitalized for a first AMI, Zhang et al., found that smoking, hypertension, and obesity were the most common conditions (38.3%) [29]. Based on a national wide population-based analysis of young patients (<55 years) with AMI, after multivariable analyses, cigarette smoking (adjusted OR 1.98; 95 CI 1.95–2.02, *p* < 0.001), obesity (adjusted OR 1.37; 95 CI 1.33–1.41, *p* = 0.003), hyperlipidaemia (adjusted OR 1.07; 95 CI 1.04–1.08, *p* < 0.001), and a family history of CAD (adjusted OR 1.35; 95 CI 1.3–1.4, *p* < 0.001) were all associated with a higher risk of developing an AMI [30]. Recent studies found that young patients (<45 years) with ACS had a greater rate of STEMI and that male sex, smoking, dyslipidaemia, and a favourable family history were the main risk factors for STEMI in these patients [31,32].

#### 3.1.5. Dyslipidaemia

LDL/HDL ratio was revealed to be a significant risk factor for AMI, which was linked to an almost fourfold increased risk of MACE. In particular, a consistent negative relationship between HDL cholesterol and CAD has been found among several cohorts of epidemiological research, with low HDL cholesterol being frequent in young people (aged 18–44 years) who present with ACS, particularly smokers [33,34]. A lower HDL-C level in a young male (aged ≤ 35 years) not only indicated a higher risk of myocardial infarction but also the severity of coronary artery lesions, with multi-vessel lesions having lower levels of HDL-C than single-vessel lesions and so indicating a negative prognosis [35]. Regarding familial hypercholesterolaemia (FH), individuals with ACS and FH are younger, have fewer comorbidities, and are more likely to be diagnosed with STEMI. A recent analysis found that type IIb hyperlipidaemia and isolated hypertriglyceridemia were linked to a more than twofold increased risk of a CV event before the age of 50 when compared to controls. The risk was lower in isolated hypercholesterolaemia (Type IIa). In addition, type IIb hyperlipidaemia was linked to a ninefold increased risk of major cardiovascular events (MACE) [36,37]. Thus, young patients with dyslipidaemia admitted for ACS should be monitored and treated aggressively during and after hospitalization.

#### 3.1.6. Gender

The presumption that there are gender-based differences in early mortality after myocardial infarction is not new. A recent cohort of young patients with ACS showed that female sex is an independent predictor of 30-day mortality, with a fivefold increased risk of death in women compared to males [38]. Furthermore, younger women (aged 65 years) with STEMI had higher in-hospital and 1-year death rates than younger males [39], and were less likely to receive the medication and revascularization suggested by guidelines during hospitalization, despite higher GRACE scores [40]. Young women with CAD have a more distinct physiology and pathology than older women and men; they are more likely to develop both an aggressive and diffuse type of CAD as they age [41]. Young women, aged 35 to 54 years, exhibited a larger comorbidity burden than young men, as well as a higher prevalence of diabetes, anaemia, obesity, peripheral vascular disease, and chronic lung disease [42]. In contrast, young patients with ACS are frequently men and have a greater incidence of smoking, dyslipidaemia, and alcohol/drug abuse [43]. Despite the differences, both men and women exhibited a significant burden of modifiable cardiovascular risk factors that could be treated both individually and as a group.

#### 3.1.7. Family History

CAD has substantial heritability and a polygenic architecture. Family history is defined as the presence of clinical CAD or sudden death in first-degree male relatives under the age of 55, or first-degree female relatives under the age of 65 [44]. Family history is an independent risk factor for MACE and ischaemic heart disease [45], with the probability of premature coronary heart disease (CHD) rising linearly as the number of afflicted family members increases [46]. Numerous studies revealed that, as compared to older persons, young patients (less than 55 years) with their first AMI were more likely to be obese, smokers, and to have a family history of early coronary artery disease [20,30,36,47,48]. Despite the link between ACS and a positive family history of CAD (FHxCAD), a large sample size research suggested that STEMI patients with FHxCAD had lower in-hospital mortality and MACEs than patients without FHxCAD, and a better long-term survival [49].

#### 3.1.8. Psychosocial and Environmental Factors

Stress and mental comorbidity enhance plaque development, instability, and rupture, although the precise pathways are still unclear [50]. A lack of social support is linked with a higher risk of developing cardiovascular diseases and a higher risk of mortality [51], with the effect of loneliness on mortality being similar to that of heavy smoking (15 cigarettes per day) [52]. The clinical presentation of CHD as well as the use of medical therapy can both be greatly impacted by psychological variables. Delay in receiving medical attention is associated with advanced age, female gender, poor socioeconomic position, denial, avoidance, and/or underestimating of the significance of heart-related symptoms of an ACS [53]. A recent meta-analysis found that those with a sedentary lifestyle had a 23% higher risk of cardiovascular mortality and a 17% higher risk of incident cardiovascular disease than those who achieved the recommended 150 min of moderate physical activity per week [54]. Physical inactivity, together with diabetes mellitus, and a family history of early coronary artery disease were found to be significant independent predictors of unexpected MI [55]. Regarding the correlation between alcohol use and ACS, Tersalvi et al., observed that heavy drinkers (>2 drinks/day) had about a 40% higher rate of in-hospital mortality and MACE (a combined outcome of in-hospital reinfarction, stroke, and/or death by any cause) than light drinkers (2 drinks/day) [56].

Additionally, Hassan et al. [57] found that patients with a history of alcoholism/dependence developed ACS 8.7 years earlier than their non-alcoholic peers. To lower early ACS morbidity and mortality, healthcare intervention in this population is necessary.

Thus, the young coronary patients have a different risk factor profile (Table 1). The characteristics of these patients are higher proportion of dyslipidaemia, family history of premature CAD and heavy smoking, and lower proportion of diabetes mellitus and arterial hypertension.

### 3.2. Lipoprotein (a)

#### 3.2.1. How, When, and What to Look For?

Lp(a) is an independent risk factor for atherosclerotic cardiovascular disease (ASCVD), valvular aortic stenosis, and mortality [58,59]. Plasma Lp(a) levels are 70% to 90% genetically determined. Other factors that play roles in Lp(a) regulation are ethnicity, race, environmental, and medical conditions [14]. Lp(a) levels are also different depending on the geographical region. An analysis performed on patients from the INTERHEART study concluded that Lp(a) concentrations >50 mg/dL were associated with an increased risk of MI (OR: 1,48; 95% CI: 1.32–1.67; *p* < 0.001). The population attributable risk of high Lp(a) for MI is highest in South Asians and Latin Americans [60].

Other important factors that play a role in Lp(a) levels are ethnicity and sex. The impact of ethnicity was reflected in multiple studies, such as the Dallas Heart Study or the ARIC study, which showed a great variability of Lp(a) levels between White and Black individuals [61]. Moreover, in the UK Biobank, median Lp(a) values decreased sequentially in Black, South Asian, White, and Chinese individuals [62]. Sex differences were also observed, with women showing Lp(a) values 5 to 10% higher than men, regardless of ethnicity. In men, Lp(a) levels tend to remain constant, whereas in women they peak during late peri- and early post-menopause [63,64].

Although the role of Lp(a) in atherosclerosis is not fully understood, it seems that Lp(a) has pro-inflammatory, pro-thrombotic, pro-atherosclerotic, and anti-fibrinolytic effects [65]. Lp(a) has a great affinity for the vascular wall, and it can be internalized and accumulated in the intima of arteries. Similar to LDL, Lp(a) promotes atherosclerotic plaque formation through various mechanisms, including expression of inflammatory cytokines (Interleukin (IL)-8, IL-6. IL-1β), expression of adhesion molecules, and monocytes chemotaxis. Thus, the atherosclerotic plaque is formed. It can be complicated by thrombosis, rupture, or erosion leading to an acute coronary syndrome. Elevated levels of Lp(a) are associated with a more complex atherosclerotic plaque and the most frequent clinical manifestation is AMI, rather than stable angina [66].

Epidemiological data show that 1 in 5 adults have lipoprotein (a) values above 150 nmol/L. These patients have a 1.5-fold risk of coronary artery disease [59]. Korneva et al., studied the relationship between Lp(a) levels and development of CHD in patients with familial hypercholesterolaemia (FH). The conclusion was that in FH patients with elevated Lp(a) levels, myocardial infarction (MI) was diagnosed 2.76 times more often [67]. In addition, elevated Lp(a) is associated with an increased risk of CAD in the absence of family history of heart disease [59].

Lp(a) appears to be the most complex lipoprotein particle, so its measurement differs and is more difficult compared to the usual lipid profile tests. Moreover, there is no clear cut-off for expressing high Lp(a) levels. Plasma concentrations vary greatly between <0.1 mg/dL and >300 mg/dL and are predominantly genetically determined, due to the variability of the LPA locus. The LPA gene is fully expressed by the age of 2 years and adult levels are generally reached by the age of 5 [68].

There are some other, less studied, factors that may influence Lp(a) concentration. Diet usually has a modest impact, and it tends to change Lp(a) levels in the direction opposite to LDL-C. Low carbohydrate/high fat diets can decrease Lp(a) by 10–15% [69]. Kidney and liver dysfunction can also lead to changes in Lp(a) values [70]. Lp(a) concentrations can be interpreted in the setting of primary or secondary prevention, with different values and interpretation methods being proposed by various studies. Although high Lp(a) concentrations correlate to an increased risk of major adverse cardiovascular events, there appears to be an important heterogeneity of studies related to Lp(a) in secondary prevention. A meta-analysis which included 18.978 individuals from 3 studies revealed a significant association of Lp(a) with the risk of CV events in patients with established coronary artery disease, but with important heterogeneity across the studies [71]. Moreover, in a study of 58 527 individuals from the Copenhagen General Population Study, Madsen et al., proposed that in the setting of secondary prevention, a reduction of Lp(a) by 50 mg/dL could reduce cardiovascular disease risk by 20% [72]. Finally, the AIM-HIGH trial revealed that Lp(a) is an important marker of residual CV risk in patients treated with statins, who already reached target LDL-C levels [73].

There is no clear threshold for Lp(a) and ASCVD risk, but rather a continuous risk increase with increasing Lp(a) concentrations [62]. However, the latest ESC/EAS Guidelines suggest that very high values (above 180 mg/dL) can help identify subjects whose lifetime ASCVD risk is equivalent to that of untreated heterozygous familial hypercholesterolaemia. Thus, the European and Canadian guidelines propose that Lp(a) concentrations should be checked at least once in every adult [1,74]. Regarding the young patients (aged < 20 years), measuring Lp(a) is recommended in individuals with genetically confirmed or clinically suspected familial hypercholesterolaemia, history of ischaemic stroke, or a parent with premature ASCVD and no other identifiable risk factors [65]. Nowadays, the general acceptance is that Lp(a) should be interpreted more as a “risk modulator”, rather than a distinct parameter, with high levels revealing individuals who might benefit from a more intensive therapeutic approach and follow-up [1].

Based on these data regarding the ASCVD of Lp(a), the most recent guidelines recommend the dosage of Lp(a) in certain situations [75] (Table 2).

#### 3.2.2. Implications of Lp(a) in Young Patients

Several studies and meta-analyses reported that elevated Lp(a) levels carry an increased risk of CAD or ischaemic stroke, and this risk is higher in the younger population [58,81]. In a prospective study that included 382 young patients (<45 years), survivors of an AMI, Rallidis et al., aimed to assess the prevalence of heterozygous familial hypercholesterolaemia (HeFH) and combined hyperlipidaemia phenotype (apolipoprotein B levels > 120 mg/dL and triglyceride levels > 170 mg/dL). Eighty-one patients had definite/probable HeFH, and sixty-two patients had the combined hyperlipidaemia phenotype. Patients with HeFH had higher levels of total cholesterol (TC), low-density lipoprotein (LDL-C), Lp(a), and apolipoprotein B [82].

In a study that aimed to analyse the association between Lp(a) levels and ACS in younger and older patients, Hanif et al., concluded that in patients younger than 45 years, serum Lp(a) (>30 nmol/L) is strongly associated with AMI [83].

Jubran et al., conducted a retrospective observational cohort study that included 134 patients under 65 years of age presenting with ACS. The aim of this study was to determine the association between elevated Lp(a) defined as >72 nmol/L (>30 mg/dL) and clinical characteristics. Elevated Lp(a) was documented in a third of the population (32%) and was associated with younger age, premature CAD (male age <55 years and female age <60 years), and previous revascularization [84].

In a descriptive and observational study, Luna et al., measured the level of Lp(a) in 159 patients with ACS. The results show that young patients (male patients under 65 years and women under 70 years) with ACS had a mean value of Lp(a) of 41.08 mg/dL. Almost 1 in 4 patients (24.5%) had Lp(a) values higher than 60 mg/dL [85].

To analyse the association between Lp(a) and premature ACS, a case-control study that included 1.457 patients with a history of ACS was conducted by Rallidis et al., Each 10 mg/dL increase in Lp(a) level was associated with a 4% increase in ACS risk in patients under 45 years of age and a 2% increase in middle-aged patients (45–60 years). They concluded that Lp(a) is an independent risk factor for ACS in individuals under 45 years of age [86].

In a cross-sectional, case-control study in India, the authors analysed the prevalence of the metabolic syndrome and the levels of Lp(a), serum ferritin, and high-sensitivity C-reactive protein (hs-CRP) in young patients (<45 years of age) with or without AMI. The hs-CRP and Lp(a) levels were significantly higher in case groups (*p* < 0.001). They concluded that these novel biomarkers may be used to assess the risk of CVD in young patients [10].

In another recent study, 130 young patients (<50 years of age) with a history of cardiovascular (CV) events were stratified into three groups according to their Lp(a) concentration: group 1: <18 mg/dL, group 2: 18–50 mg/dL, group 3: >50 mg/dL. Overall, 69% of patients had Lp(a) concentration above 50 mg/dL. The results showed that Lp(a) is one of the most important risk factors for cardiovascular events in young patients [87].

All these studies highlight the role of lipoprotein (a) as an important risk factor for coronary artery disease, especially in young patients with AMI (Table 3). Hence, the recent guidelines and consensus statements [74,88] have introduced the recommendation to measure the lipoprotein (a) values in high-risk patient groups, particularly in young patients with an acute coronary syndrome.

#### 3.2.3. Therapeutic Approaches

At the moment, there are no specific drugs approved for lowering Lp(a) concentrations. Proprotein convertase subtilisin/kexin type 9 (PCSK9) inhibitors have been shown to lower Lp(a), as well as LDL-C levels. An analysis of the ODYSSEY Outcomes trial, which included patients with a recent acute coronary syndrome, revealed that PCSK9 was successful in reducing lipoprotein (a) levels and that reduction independently contributed to subsequent MACE reduction [89].

Studies regarding statins and Lp(a) are inconclusive. Although they successfully reduce LDL-C levels, statins have been shown to increase Lp(a) concentrations through mechanisms which are not fully understood yet. A recent meta-analysis, which included 24 448 individuals, did not find clinically important differences in Lp(a) concentrations in statin-treated patients [90].

There are some studies underway which follow specific Lp(a) lowering therapies. One of these potential therapies are antisense oligonucleotides that inhibit the hepatic production of apo(a) mRNA. In a trial that included 286 participants with elevated Lp(a) levels and established cardiovascular disease, AKCEA-APO(a)-LRx was shown to successfully reduce Lp(a) concentrations in a dose-dependent manner [91].

Another phase 2 trial designed to evaluate the efficacy and safety of olpasiran, a small interfering RNA molecule that prevents assembly of the lipoprotein (a) particle in the hepatocyte, was recently published. In this study, olpasiran led to a significant and sustained reduction in the Lp(a) concentration, when administered every 12 weeks in patients with established atherosclerotic cardiovascular disease and a Lp(a) concentration of more than 150 nmol per litre [92].

Therefore, in the absence of specific lowering therapies for patients with elevated levels of Lp(a), it is essential to provide integrated management of them, with an effective control of all cardiovascular risk factors. In addition, lipoprotein aphaeresis can be a solution in patients with progressive cardiovascular disease and very high Lp(a), despite optimal management of cardiovascular risk factors [1].

## 4. Conclusions

Although there are important advances in the management of “young” patients with acute myocardial infarction, this pathology still remains one of the most important causes of death worldwide. The results from the ongoing Lp(a) HORIZON study are crucial in order to understand the impact of Lp(a) lowering therapy on major cardiovascular events in patients with established CVD. This is a randomized double-blind, placebo-controlled, phase 3 multicentre trial with Pelacarsen (TQJ230) 80 mg injected monthly and administered subcutaneously, and the estimated study completion date is 2025. The impact of olpasiran on major cardiovascular events in patients with atherosclerotic cardiovascular disease and elevated lipoprotein (a) is currently being studied in a phase 3 trial which is expected to be completed by the end of 2026. Thus, we consider that the future holds great promise in terms of standardization of Lp(a) measurement and specific Lp(a)-lowering therapies that will improve the clinical outcomes of “young” patients with AMI.

The Incidence of acute myocardial infarction in “young” patients is increasing. There is clear evidence that elevated Lp(a) is an important risk factor for atherosclerotic cardiovascular disease, especially in young patients and those with familial hypercholesterolaemia. Furthermore, recent research in terms of the standardization of Lp(a) measurement and specific Lp(a)-lowering therapies might be able to guide more individualized management of “young” patients with AMI, in order to improve their outcomes.

## Figures and Tables

**Table 1:** Traditional cardiovascular risk factors in “young” vs. “old” patients with myocardial infarction.

Traditional Cardiovascular Risk Factor	“Young” Patients	“Old” Patients
Arterial hypertension	Reduced prevalence of hypertension in younger STEMI patients (<45 years), compared to older ones [14] The most important risk factor for recurrent cardiovascular events in the younger group [15] Independent risk factor for multivessel disease in younger ACS patients (≤45 years) with a poor outcome [17]	Older STEMI patients (>45 years) had higher prevalence of arterial hypertension [14] Untreated hypertension was lower in those between 55 and 70 years compared to younger patients (<55 years) [16]
Smoking	In STEMI patients, smoking was shown to be the most prevalent risk factor (<45 years) [19] Strong predictor of future MI in younger patients [20] Young AMI patients (18-44 years) had higher rates of smoking [21,22]	Rates of current smokers were lower in the older CAD group (>65 years) [20] The correlation of water pipe smoking and first AMI was lower in the older group (>45 years) compared to younger one [22]
Diabetes mellitus	Type 2 DM was one of the common risk factors in young AMI patients (≤40 years) [25] Diabetes was linked to increased long-term all cause and cardiovascular mortality in younger AMI patients (<50 years) [26] The strongest predictor of ACS in women ≤ 45 years of age, with a sixfold increase in risk [28]	In older ACS women (63-64 years) diabetes was more prevalent than in the younger group (<45 years) [28]
Obesity	Obesity was along with cigarette smoking, hyperlipidaemia, and family history of CAD associated with a higher risk of developing AMI in young patients (<55 years) [30]	Older patients with AMI (>40 years) presented a lower body weight compared to the younger group (mean 79.7 kg vs 85.9 kg) [31]
Dyslipidaemia	Low HDLc was frequent in young smokers ACS people (18-44 years) [34] Type IIb hyperlipidaemia and isolated hypertriglyceridemia were linked to a more than twofold increased risk of a CV event (<50 years)	Older individuals are more likely to take statins, because of past cardiovascular disease, thus presenting lower levels of LDLc compared to younger one [33]
Gender	Female sex is an independent predictor of 30-day mortality with a fivefold increased risk of death compared to male in younger patients [38] Young women (35-54 years) presented a larger comorbidity burden than young men [42]	Women with AMI and ≥65 years presented a higher readmission rate compared to male ones [43]
Family history	Young AMI patients (<55 years) were more likely to have a family history of early CAD [20,30,36,47,48]	Older patients (>50 years) presented less family history of CAD compared to younger ones (21.4% vs 10.4%) [47]

AMI, acute myocardial infarction; ACS, acute coronary syndrome; CAD, coronary artery disease; CV, cardiovascular; DM, diabetes mellitus; HDLc, high-density lipoprotein cholesterol; LDLc, low-density lipoprotein cholesterol; MI, myocardial infarction; STEMI, ST-elevation myocardial infarction.

**Table 2:** Recommendation regarding the use of Lp(a) measurements in clinical practice.

Guidelines	Recommendation
ESC/EAS (European Society of Cardiology/European Atherosclerosis Society) for the management of dyslipidemias [76]	Family history of premature cardiovascular disease Once in the lifetime of each adult person Moderate to high risk of cardiovascular event
HEART UK (Hyperlipidaemia Education and Atherosclerosis Research Trust UK) [77]	Calcific aortic valve stenosis Familial hypercholesterolaemia or other genetic forms of dyslipidaemia First-degree relatives of those with high Lp(a) levels (>200 nmol/L) Personal or family history of premature ASCVD (<60 years of age) A borderline increased 10-year risk of cardiovascular events
AHA/ACC (American Heart Association/American College of Cardiology) Guideline of the Management of Blood Cholesterol [78]	Personal or family history of premature ASCVD
NLA (National Lipid Association) [79]	Premature ASCVD Recurrent or progressive ASCVD
CCS (Canadian Cardiovascular Society) [80]	Family history of premature coronary artery disease Individuals with intermediate risk scores

ASCVD, atherosclerotic cardiovascular disease; Lp(a), lipoprotein (a).

**Table 3:** Studies regarding the implications of lipoprotein (a) in myocardial infarction in the “young”.

Study	Country	Population	Measurement	High Lp(a) Threshold	Methodology	Results
Afshar et al. [8]	USA	2606 participants from the Framingham Offspring Cohort (median age of 54, 45% men)	ELISA	≥100 nmol/L	Prospective cohort study with a median follow-up of 15 years	Elevated levels of Lp(a) were associated with a high incidence of cardiovascular disease
Rallidis et al. [82]	Greece	382 participants ≤ 40 years who suffered an AMI	High sensitivity particle-enhanced immunonephelometry	apoB > 120 mg/dL	Retrospective cohort study	Lp(a) levels were higher in patients with combined hyperlipidaemia phenotype
Hanif et al. [83]	Pakistan	180 participants with ACS (70% male) subdivided into 45 cases aged ≤ 45 years (considered “young”), respectively, 45 aged > 45 years, each group with age-matched healthy controls	Lp(a) assay on Abbott Architect platform ci8200 using a Latex enhanced technique	High risk for ACS: 31–50 mg/dL, very high risk for ACS: >50 mg/dL	Case control study	In young patients, Lp(a) levels were strongly associated with coronary vascular disease, even with comparative values of LDL and HDL between cases and controls
Jubran [84]	Israel	134 patients ≤ 65 years, with a mean age of 52 ± 8 years, 83% male, presenting with ACS	Particle enhanced trubidimetric immunoassay	>30 mg/dL	Retrospective observational cohort analysis	Elevated Lp(a) levels were independently associated with CAD in young and middle-aged patients
Luna et al. [85]	Spain	159 patients (men under 65 years and women under 70 years), who suffered a STEMI or NSTEMI	ELISA	>60 mg/dL	Descriptive and observational study	Mean Lp(a) levels were 41.08 mg/dL, and 24.5% of patients had values greater than 60 mg/dL
Rallidis et al. [86]	Greece	1457 patients with a history of ACS (median age 54.8 ± 13 years, 86% male) and 2090 age- and sex-matched controls with no CAD	High sensitivity particle-enhanced immunonephelometry	>30 mg/dL	Case-control study	In young patients (<45 years), Lp(a) is an independent risk factor for ACS and elevated Lp(a) levels increase this risk threefold; the correlation was not as strong in the 45–60 years category and was not observed at all >60 years
Schatz et al. [87]	Germany	130 patients with a history of cardiovascular events, aged ≤ 50 years	Immune nephelometry	Three groups: <18 mg/dL, 18–50 mg/dL, >50 mg/dL	Prospective observational study	Lp(a) levels >50 mg/dL were associated with premature CAD
Finneran et al. [59]	UK	153 228 patients without prevalent CAD and no family history of heart disease, with a mean age of 58.4 years, of which 52.2% were female with follow-up time of <9 years	Immunoturbidimetric assay	>150 nmol/L	Prospective, observational cohort study	In patients with no personal or familial (first degree) history of CAD, high Lp(a) levels were associated with an increased risk of incident CAD
Korneva et al. [67]	Karelia Republic	81 patients (middle age was 39.1 ± 0.4 years, 33 males, 48 females), with familial hypercholesterolaemia	Immunoturbidimetry	>30 mg/dL	Prospective observational study	Elevated Lp(a) levels were associated with an increased risk of CAD in patients with familial hypercholesterolaemia

ACS, acute coronary syndrome; AMI, acute myocardial infraction; CAD, coronary artery disease; ELISA, enzyme-linked immunosorbent assay; HDLc, high-density lipoprotein cholesterol; LDLc, low-density lipoprotein cholesterol; Lp(a), lipoprotein (a); NSTEMI, non-ST-elevation myocardial infarction; STEMI, ST-elevation myocardial infarction.

## Data Availability

Not applicable.
